# Evidence-based clinical use of insulin premixtures

**DOI:** 10.1186/1758-5996-5-50

**Published:** 2013-09-06

**Authors:** Marcos Antônio Tambascia, Márcia Nery, Jorge Luiz Gross, Mariana Narbot Ermetice, Carolina Piras de Oliveira

**Affiliations:** 1 Faculty of Medical Sciences, State University of Campinas, Brazil Rua Frei Manoel da Ressurreição 965, Campinas, SP, Brazil; 2 Diabetes Unit - Endocrinology and Metabolism Service, Clinical Hospital of Faculty of Medicine, University of São Paulo, São Paulo, Brazil; 3 Department of Internal Medicine, Faculty of Medicine of Federal University of Rio Grande do Sul, Rio Grande do Sul, Brazil; 4 Diabetes Group, Eli Lilly do Brazil São Paulo, São Paulo, Brazil; 5 Currently at Novo Nordisk Brazil, São Paulo, Brazil

**Keywords:** Type 2 diabetes mellitus, Insulin, Premixed, Lispro, Glycated hemoglobin, Treatment goals, Titration

## Abstract

Brazil is expected to have 19.6 million patients with diabetes by the year 2030. A key concept in the treatment of type 2 diabetes mellitus (T2DM) is establishing individualized glycemic goals based on each patient’s clinical characteristics, which impact the choice of antihyperglycemic therapy. Targets for glycemic control, including fasting blood glucose, postprandial blood glucose, and glycated hemoglobin (A1C), are often not reached solely with antihyperglycemic therapy, and insulin therapy is often required. Basal insulin is considered an initial strategy; however, premixed insulins are convenient and are equally or more effective, especially for patients who require both basal and prandial control but desire a more simplified strategy involving fewer daily injections than a basal-bolus regimen. Most physicians are reluctant to transition patients to insulin treatment due to inappropriate assumptions and insufficient information. We conducted a nonsystematic review in PubMed and identified the most relevant and recently published articles that compared the use of premixed insulin versus basal insulin analogues used alone or in combination with rapid-acting insulin analogues before meals in patients with T2DM. These studies suggest that premixed insulin analogues are equally or more effective in reducing A1C compared to basal insulin analogues alone in spite of the small increase in the risk of nonsevere hypoglycemic events and nonclinically significant weight gain. Premixed insulin analogues can be used in insulin-naïve patients, in patients already on basal insulin therapy, and those using basal-bolus therapy who are noncompliant with blood glucose self-monitoring and titration of multiple insulin doses. We additionally provide practical aspects related to titration for the specific premixed insulin analogue formulations commercially available in Brazil.

## Introduction

In 2011, the prevalence of diabetes mellitus worldwide was approximately 366 million people [1]. In Central and South America, it has been estimated that 25.1 million people have diabetes [1]. Approximately 12.4 million adults (20–79 years of age) in Brazil had diabetes in 2011; this is expected to increase to 19.6 million by 2030 [1]. In keeping with this epidemic, there have been a growing number of cases in younger individuals with longer life expectancies, many of whom will require treatment with exogenous insulin [1].

The United Kingdom Prospective Diabetes Study (UKPDS) comparing intensive treatment with conventional treatment in patients with type 2 diabetes mellitus (T2DM) showed that appropriate glycemic control significantly reduces the rate of microvascular complications, such as retinopathy, nephropathy, and neuropathy [2], which persisted in the long-term follow-up to this study [3]. Appropriate blood glucose (BG) control is also important for cardiovascular disease (CVD) [4], although the reduction of CVD requires a multifactorial approach: control of blood pressure, lipid profile, and body weight; smoking cessation; and regular aerobic exercise [5].

In order to control BG levels, medical associations, such as the American Diabetes Association (ADA), the European Association for the Study of Diabetes (EASD), the American Association of Clinical Endocrinologists (AACE), the American College of Endocrinology (ACE), and the Brazilian Diabetes Society, recommend that diet and lifestyle changes, increased physical activity, and treatment with metformin be implemented soon after diagnosis. Usually there is an improvement of glycemic control for a period of time, depending on the patient’s age, clinical condition, comorbidities, etc., but if it still remains inadequate or there is progressive deterioration, then the addition of other antihyperglycemic agents (AHAs) is usually necessary [6-11]. The combination of 2 or 3 noninsulin AHAs with different mechanisms of action is frequently used, since most patients are reluctant to start insulin. Moreover, patients with T2DM are sometimes able to maintain endogenous insulin secretion even in later stages of the disease [12]. If a patient has not reached the desirable glycemic control goals or presents signs that are related to low insulin action, such as catabolic features (e.g., weight loss and/or ketonuria) or persistent high levels of BG (>300-350 mg/dL), then insulin therapy is recommended [8]. Several strategies for insulin therapy in addition to oral AHAs can be used: neutral protamine Hagedorn (NPH) insulin, basal insulin analogues (detemir or glargine), or premixed insulins.

Recent studies suggest that premixed insulin analogues are more effective in reducing glycated hemoglobin (A1C) than basal insulin analogues used alone [13-19]. However, premixed analogues tend to cause a small increase in the risk of nonsevere hypoglycemic events and a small, not clinically significant weight gain [13-19]. In general, premixed insulin analogues are options for providing basal and prandial insulin in a simple, less complicated way than the basal-bolus approach, and they can be used in insulin-naïve patients. Advantages of this regimen include the need for fewer BG measurements during the day while ensuring that the patient receives both basal insulin and bolus insulin at meals with 2 or a maximum of 3 injections per day [13,20].

The aim of this nonsystematic overview is to discuss barriers to insulin initiation, individualized treatment goals, and recent studies that compared the use of premixed insulin versus basal insulin analogues used alone or in combination with rapid-acting insulin analogues before meals in patients with T2DM. It also aims to provide practical aspects of insulin treatment that can be used to encourage and motivate patients to comply with treatment and ultimately attain desirable glycemic control.

A comprehensive literature search was conducted in PubMed (1995 to 2012) using the search terms: type 2 diabetes mellitus, insulin, premixed, lispro, glycated hemoglobin, biphasic insulin aspart, treatment goals, targets, and titration, to identify the most relevant and recently published English language articles, including reviews, meta-analyses, randomized trials, and treatment guidelines related to these topics.

### Barriers to insulin initiation

Most insulin-naïve patients and their physicians are reluctant to transition to insulin treatment [21]. The reluctance to begin insulin treatment comes from the perception that the treatment regimen is complex; however, the decision to start insulin may be based on insufficient information. In a survey of insulin-naïve patients with poorly controlled T2DM, who had been treated with 2 or more oral agents and were recently prescribed insulin, the authors observed that those failing to initiate prescribed insulin commonly reported misconceptions and concerns regarding insulin risk (35% of the patients believed that insulin causes blindness, renal failure, amputations, heart attacks, strokes, or early death), injections (e.g., injection phobia), hypoglycemia, and impact on social life and work [22]. Nonadherent patients frequently felt their health care provider had not adequately explained the risks and benefits of insulin. Insulin adherence may be improved through better provider communication regarding risks, shared decision making, and insulin self-management training.

Intensification of insulin therapy is often required in patients with T2DM. The standard for intensive care consists of the combination of basal insulin with a preprandial insulin (rapid-acting) basal-bolus. However, premixed insulin analogues are more convenient and equally or more effective, despite being potentially associated with slightly greater weight gain and nonsevere hypoglycemic reactions [7,10,11,13,23-26].

### Treatment goals per patient profiles

A key concept in the treatment of T2DM is establishing individualized glycemic goals [9]. Targets for glycemic control, including fasting BG (FBG), postprandial BG, and A1C as well as lipid and blood pressure levels, should be identified by the physician and the patient and ultimately achieved by therapeutic means. A1C reflects average BG over the previous 3 months [27], and it is a strong predictor of diabetic microvascular complications (retinopathy, neuropathy, and nephropathy) [28,29]. The baseline A1C in patients with T2DM guides the physician in establishing the initial therapeutic approach and is also used as a reference for adjusting the therapy thereafter.

The A1C treatment target is established by taking into account the balance needed between the level of BG control, the patient’s risk of chronic complications, the risk of treatment-related adverse events, and patient-related psychosocioeconomic factors [9]. Thus, routine check-ups are recommended twice a year for patients meeting treatment goals and quarterly (every 3 months) for all patients with T2DM whose therapy has been recently modified or who are not meeting their glycemic goals [7,10,11,30].

The role of FBG and postprandial BG in hyperglycemia remains controversial. A1C is a function of FBG and postprandial BG, but the percentages and conditions under which each of these components contributes to A1C values have remained largely unknown. A study by Monnier et al. [31] suggests that postprandial BG is the main contributor to the metabolic disequilibrium in those patients with mild or moderate hyperglycemia. In contrast, FBG is the main contributor to daytime hyperglycemia in those patients with poor glycemic control (A1C >8.4%) [31]. Table 1 presents suggested values for FBG and postprandial BG and their corresponding A1C levels based on the results of this study. A recent study [32] found that FBG area under the concentration-time curve (AUC) predominated at all A1C quartiles and that there were lower decreases in FBG and FBG AUC in patients above the A1C target. This finding suggests that patients required a higher insulin dose or needed to be switched to a more intensive treatment.

**Table 1:** **Values of mean, fasting, and postprandial blood glucose (BG) corresponding to glycated hemoglobin (A1C)**[31]

**A1C (%)**	**Mean BG (mg/dL)**	**Fasting/preprandial BG (mg/dL)**	**Postprandial BG (mg/dL)**
6.0	126	100	140
6.5	140	110	150
7.0	154	110	160
7.5	168	120	180
8.0	183	130	200

Another recent study [33], which reviewed strategies of intensification of glucose control with different treatment options for T2DM, concluded that the type of treatment used to achieve glycemic control can focus on FBG or postprandial BG. These findings suggest that if lowering FBG is the treatment goal (basal insulin), then FBG would contribute to approximately 34% of A1C (mean value 7.7%) and postprandial BG to approximately 66% of A1C. If lowering postprandial BG is the treatment goal (intensifying insulin treatment with premixed insulin or adding prandial insulin), then FBG would contribute to approximately 68% of A1C (mean 7.7%) and 32% of postprandial BG [33]. Thus, it is important to bear in mind that in order to normalize glycemic exposure, attention must be paid to both basal and postprandial glucose excursions.

Due to the results obtained from the UKPDS, the ADA and AACE recently agreed that the glycemic target for most patients with T2DM should be A1C ≤6.5% or ≤7% [2,7,11]. However, recent studies have found that intensive treatment does not lead to a reduction in new CVD or reduced mortality in patients with a prior history of CVD [34,35]. In fact, the Action to Control Cardiovascular Risk in Diabetes (ACCORD) trial reported increased mortality in patients with T2DM over an average of 10 years and an increase in several risk factors for CVD when patients were treated with the objective of maintaining A1C levels close to the reference values [36]. Thus, an important consideration is how to balance the potential benefits of intensive control with the risk of such intensive targets. In that context, patients’ clinical and psychosocial characteristics are key factors in the modification of the glycemic targets.

Riddle [37] recently commented on the clinical implications stemming from the ACCORD trial results. The main question is how to determine which patients are at greater risk of death if undergoing intensive treatment. It appears that patients with A1C values >8.5% and those with limited A1C-lowering response after the first 12 months of treatment may be at higher risk. Riddle concluded that, based on the ACCORD study results, glycemic targets should be individualized and current therapies adapted to each patient’s needs.

### Clinical conditions that might impact the choice of insulin therapy

In order to set glycemic goals for the management of hyperglycemia in patients with T2DM, the treating physician must consider various important patient-specific aspects that will impact the choice of insulin therapy, such as age, presence of comorbidities, duration of diabetes, presence of microvascular disease, history of severe hypoglycemia, and psychosocial and economic contexts. The recently published ADA and EASD [8] position statement also reinforces this strategy as a good practice when dealing with T2DM in naturalistic clinical practice as part of individualizing therapy for the patient.

### Premixed insulin

Premixed analogues provide both basal and postprandial coverage starting with 1 injection, but they are generally administered twice daily (BID), 1 injection at breakfast and 1 at supper. Physicians may recommend adding further injections, depending on patient’s individual needs [23,38-41].

In Brazil, there are currently 2 types of premixed insulins available: human insulin and analogue insulin (Table 2). Premixed human insulin consists of a basal component of human insulin (70%) and a prandial component of unmodified regular human insulin (30%) (Humulin® 70/30, Eli Lilly and Company, Indianapolis, Indiana, USA; Novolin® 70/30, Novo Nordisk, Bagsvaerd, Denmark). Premixed insulin analogues have 2 components in their formulation: the prandial component is a rapid-acting analogue (either insulin lispro or aspart), and the basal component is insulin lispro or aspart protamine suspension. The rapid-acting analogue is also derived from recombinant DNA, but with an amino acid modification to make its action rapid, and an intermediate-acting analogue derived from the addition of protamine. These include insulin lispro mix 25 (LM25) (Humalog® Mix25™, Eli Lilly and Company, Indianapolis, Indiana, USA), insulin lispro mix 50 (LM50) (Humalog® Mix50™, Eli Lilly and Company, Indianapolis, Indiana, USA), insulin aspart 70/30 (BIAsp 30) (NovoMix® 30, Novo Nordisk, Bagsvaerd, Denmark), and insulin aspart 50/50 (NovoMix® 50, Novo Nordisk, Bagsvaerd, Denmark) (Table 2).

**Table 2:** Insulin premixture formulations

**Manufacturer**	**Commercial name**	**Composition**
Eli Lilly and Company	Humulin® 70/30	70% human insulin isophane suspension, 30% regular human insulin
	Humalog® Mix25™	25% insulin lispro, 75% insulin lispro protamine suspension
	Humalog® Mix50™	50% insulin lispro, 50% insulin lispro protamine suspension
Novo Nordisk	Novolin® 70/30^*^	70% human insulin isophane suspension, 30% regular human insulin
	NovoMix® 30	70% protamine-crystallized insulin aspart and 30% insulin aspart
	NovoMix® 50^*^	50% protamine-crystallized insulin aspart and 50% insulin aspart

*Not available commercially in Brazil but still available in many other countries; approved by the U.S. Food and Drug Administration and the European Medicines Evaluation Agency.

Premixed insulin analogues do not have 2 distinct peaks [26,42-44]. Once absorbed, the time of action corresponds to the rapid onset of action, and the duration of action corresponds to the intermediate insulin component. The peak of action is unimodal, corresponding to the maximum effect of rapid insulin, and it is steep with a slow decline [42-44]. Their rapid absorption due to the rapid component and the more pronounced onset of insulin action means that these analogues can be dosed immediately before or following a meal [26,43,44].

### Glycemic monitoring

#### Using capillary glucose monitoring to adjust doses of premixed insulin

Measurement of capillary glucose levels performed by patients themselves (self-monitoring) is an important tool in assessing BG levels and making the required adjustments to treatment to facilitate improved glycemic control [45]. This helps the patients to better understand the varying effects of nutrition, exercise, and stress on their glycemic control [46]. It also allows patients to detect hypoglycemia and to take corrective measures, such as an insulin dose adjustment [46]. The required frequency of self-monitoring depends on the patient’s disease state, the treatment regimen, the target level of glycemic control to be achieved, and physician judgment [47]. There are treatment regimens, such as those involving the use of premixed insulin analogues, that allow less frequent BG self-monitoring [7]. Various limitations of effective home-based BG self-monitoring should be taken into consideration. The major obstacles for effective self-monitoring are the general lack of knowledge and training of both patients and health care professionals regarding its usefulness, appropriate technique, correct interpretation of the data, appropriate medication adjustments and diet or physical activity adjustments, as well as the effect that BG self-monitoring can have on disease-complication onset [7]. The Brazilian government aids patients with diabetes by providing the equipment and supplies (glucometer and glucose test strips) needed for BG self-monitoring for patients who are using insulin [48]. Therefore, comprehensive education programs for health care professionals, patients, and patients’ support networks (family, close friends, and caregivers) can help to overcome existing obstacles.

#### First step: preprandial monitoring

When initiating treatment with premixed analogue insulin BID, it is recommended that patients measure BG just before breakfast and dinner as well as when there are symptoms consistent with hypoglycemia [13]. However, if the patient has a snack midafternoon, it is recommended to measure BG before the snack instead of before dinner because the carbohydrate content of the snack will influence BG levels before dinner. When glycemic control is achieved, measurements of BG can be performed less frequently (preferably before breakfast, 1–2 times a week) to confirm that adequate glycemic control is being maintained. Approximately 3 months after reaching BG objectives, measurement of A1C is recommended [7,10].

#### Second step: postprandial monitoring

In cases in which the established treatment goal has not been achieved, it is suggested that paired measurements of BG before and after main meals be performed to define when the use of corrective dietary measures and/or a larger dosage of insulin is required. Whenever optimal glycemic control is achieved, the patient might perform less frequent BG measurements. It is important to note that patients can achieve lower postprandial BG concentrations by reducing high glycemic index carbohydrates (potatoes, white rice, white bread, white flour, white pasta, polenta, etc.) in their diets [49].

### Premixed insulin analogues

It is unclear which patients would most benefit from the use of premixed analogues, but based on data from a meta-analysis by Giugliano et al. [50] and the DURAbility of Basal versus LM25 insulin Efficacy (DURABLE) study [51], it is possible to infer that certain patients would benefit more by starting insulin treatment with premixed insulin analogues. Such patients include those who have A1C around 8.5%, have higher elevations in postprandial glucose (difference between preprandial and postprandial >50 mg/dL), are less capable or willing to perform several measurements of self-monitored BG during the day, and are able to eat regularly [51].

Whereas LM25 can be used for patients who have not reached adequate postprandial BG concentrations (13,14,52), LM50 can be used to enhance premixed insulin treatments and is a good choice for patients who are already using premixed analogues [53]. LM50 can also be an option for those patients using basal insulin with multiple doses of rapid insulin but who are not compliant with the treatment regimen [20,54]. This higher premixed insulin can also be an initial choice of treatment for patients who may need higher daily doses of rapid insulin or those who eat meals with a high carbohydrate content [18,53].

### Indications to use premixed insulin analogues

#### Insulin-treatment-naïve patients

It is well established that patients with T2DM who do not achieve glycemic goals despite lifestyle changes, increased physical activity, and use of one or more AHAs should start treatment with insulin [8,9]. The introductory type of insulin used depends on the degree of insulin resistance and the amount of food consumed at each meal [7,11,42]. In addition, recent evidence suggests that LM25 provides similar glycemic control to that of insulin lispro plus glargine in insulin-naïve patients with uncontrolled diabetes on AHAs [55]. Noninferiority of insulin LM25 was demonstrated on a 0.4% margin for glycemic control achieved with intensifying therapy in such a patient group [55].

Once the physician has opted for a premixed insulin, such as LM25 or BIAsp 30, insulin can be administered initially at a dose of 10 U or 0.1 U/kg before breakfast. The dose could then be titrated or repeated before dinner on the following days, depending on individual needs and eating patterns. Titration of insulin before breakfast is based on BG before dinner, and titration before dinner is based on BG before breakfast, as described in Table 3. Insulin is adjusted every 3 days, according to the average BG levels at the respective times [56].

**Table 3:** **Algorithm for titration of premixed insulin‡**[56]

**Preprandial blood glucose (mg/dL)**	**Dose adjustment**
<80	↓ by 2
80-109	Maintain dose
110-139	↑ by 2
140-179	↑ by 4
≥180	↑ by 6

‡This algorithm targets preprandial glucose ≤110 mg/dL to achieve a target A1C <7%, and can be used for patients using premixed insulin twice daily (BID) and 3 times daily (TID), including 3-day average blood glucose measurements.

If BID - Adjust morning insulin dose based on the average predinner blood glucose and evening insulin dose based on the average prebreakfast/fasting blood glucose.

If TID - Adjust morning insulin dose based on prelunch glucose levels, lunch insulin dose based on predinner glucose levels, and evening insulin dose based on prebreakfast/fasting glucose level.

Do not increase dose in case of hypoglycemia (blood glucose <70 mg/dL) or its symptoms.

If A1C is not below the pre-established goals at 4 months after insulin initiation, despite appropriate preprandial BG, then postprandial BG should be assessed. In these cases, if the patient does not receive insulin before lunch (as with a BID regimen), it is particularly important to measure BG 2 hours after lunch, as high values may indicate the need to add a third dose of insulin before this meal. Rapid-acting insulin or even an additional dose of premixed insulin may be indicated if BG before dinner is also above target goals (the choice of the rapid proportion will depend on the amount of carbohydrates consumed in this meal) [49].

For patients whose main meal is dinner, a premixed insulin analogue can be initiated with a single dose of 10 U or 0.1 U/kg before dinner followed by an evaluation of the effect on BG before breakfast. When the goal is achieved, the insulin dose is maintained and A1C is assessed after 3 to 4 months. If A1C is not within the desired range, BG is measured before dinner for a few days. If the values are high, premixed insulin analogues can be initiated before breakfast [10].

#### Patients already on basal insulin therapy

Some patients taking 1 daily dose of basal insulin (NPH, detemir, or glargine) may not achieve the proposed A1C targets due to postprandial hyperglycemia despite appropriate fasting BG levels. In such cases, a prandial insulin can be added or premixed analogues can be used. If the patient was using basal or basal plus prandial insulin, then the total daily insulin dose of the premixed analogue can be given on a 1:1 basis, thus providing the same total daily dose as before, with half the dose administered before breakfast and the other half before dinner [56]. There are different compositions of premixed insulin options, depending on the amount of food consumed in each meal. The titration process is as previously described (Table 3). If needed, glycemic control may be improved with premixed insulin 3 times a day [8]. Premixed insulin for patients already on basal insulin may offer greater capability to reduce A1C as it would provide both fasting and prandial glucose coverage with just 2 or 3 injections, whereas basal plus prandial injections administered as separate formulations may require more injections and increase the need for BG self-monitoring.

However, we stress the fact that treatment needs to be individualized based on the results of a recent comparison of BIAsp 30 with insulin glargine in patients with T2DM who were not achieving glycemic targets on basal insulin or AHAs [57]. Findings of a large improvement in the glargine group suggest that remaining on basal insulin and truly optimizing the dose might be equally beneficial for some patients.

#### Patients using basal-bolus insulin who are noncompliant with BG self-monitoring and titration of multiple insulin doses

For patients on intensive insulin treatment but who are noncompliant due to treatment complexity, do not follow BG self-monitoring, or do not feel confident with titration of multiple insulin doses, the use of thrice-daily (TID) premixed insulin, with a balanced composition of rapid and intermediate or longer-acting insulin, is the most appropriate strategy [25]. BG self-monitoring for an intensive treatment, such as basal-bolus insulin, includes at least 6 measures a day (before and after the most important meals), which could be more, depending on the amount of carbohydrate of an extra meal during the day [8,10]. To enable adequate titration of premixed insulin, the recommendation for BG self-monitoring is one measurement taken before each meal injection [14,20]. As seen in the study by Rosenstock et al. [20], although the noninferiority of premixed insulins versus basal-bolus therapy was not demonstrated, a larger proportion of patients treated with basal-bolus therapy achieved A1C targets of <7% (54% versus 69%, p = 0.009) and ≤6.5% (35% versus 50%, p = 0.01). For patients who have not achieved fasting BG target and are not able to increase the dose of premixed insulin before dinner due to hypoglycemia after dinner, it is suggested that they replace the dinner dose of LM50 with a premixed insulin containing a less rapid-acting component, and maintain the same concentration of rapid and basal insulin before breakfast and lunch if the goals for those time points have been achieved [18,20].

### Titrating premixed analogue insulins

According to major studies performed in patients with T2DM using insulin premixtures [20,53,54,58-62], the titration schedule should take into account the number of injections that the patient is receiving. In patients with T2DM treated with LM25 BID, the dose should be titrated every 3 to 4 days (twice a week), as shown in Table 3. Patients treated with LM50 who are usually receiving TID doses should adjust the premixed insulin dose every 3 to 4 days (twice a week), as shown in Table 4.

**Table 4:** **Algorithm for premixed insulin titration – adjustment according to postprandial glucose**[54]

**Postprandial blood glucose (mg/dL)**	**Dose adjustment (units)**
144-179	↑ by 1 unit
180-218	↑ by 2 units
219-258	↑ by 3 units
≥259	↑ by 4 units

Postprandial adjustment: Adjust insulin dose administered in a given meal according to the 3-day average capillary blood glucose after the meal; postprandial was defined as <2 hours after the meal with a blood glucose goal of <144 mg/dL.

When experiencing severe or recurrent hypoglycemia early in the morning, after breakfast, or before dinner with a TID premixed insulin analogue regimen, or after breakfast and before lunch with a BID regimen, the premixed insulin dose received before the hypoglycemic event should be reduced by 10%-20%. Treatment regimens of patients on concomitant AHAs should be handled with caution because adjustments in AHA doses may be required to reduce the incidence of hypoglycemia when using premixtures [20,63]. To achieve better glycemic control for patients who require a more tailored approach, physicians may prefer to titrate the premixed insulin according to the postprandial BG measure to achieve a postprandial BG goal of <144 mg/dL (Table 4) [54].

### Managing oral antihyperglycemic agents

Patients treated with metformin are monitored in order to detect increasing levels of creatinine. Typical contraindications for metformin use are kidney, heart, liver, and lung failure. Secretagogue agents, such as sulfonylureas and glinides, can be maintained until a major secretory failure develops and should be discontinued when prandial insulin is introduced, since postprandial insulin can usually be managed with a rapid-acting insulin analogue or a premixed insulin preparation [63]. This usually occurs when the patient has adequate BG concentrations at night and early morning, but experiences daytime hyperglycemia, in which case treatment with insulin only is recommended [10].

### How to manage hypoglycemia and weight gain

One of the main limitations of insulin therapy and the attainment of glycemic control is the weight gain associated with insulin treatment. It has been widely observed in numerous human insulin and premixed insulin studies [64-66] and can seriously affect patient compliance with treatment and thus, may adversely affect the prognosis. In the UKPDS study [3], patients in the intensive treatment cohort gained approximately 5 kg more than those in the conventional treatment cohort during a 10-year follow-up period. Likewise, in other studies evaluating intensive therapy versus conventional therapy [34-36], weight gain was generally greater in patients treated with intensive therapy. The weight gain associated with intensive therapy and improvement in glycemic control has been attributed to multiple factors, such as the anabolic effect of insulin itself, reduction of glycosuria (conservation of glucose calories previously excreted by the kidneys), reduced catabolism rate, appetite increase, hypoglycemia therapy, and defensive feeding to prevent hypoglycemia or simply due to the fear of hypoglycemia [64,66,67].

In order to control weight gain, several strategies can be employed. A reduction in insulin dose requirements by increasing the patient’s sensitivity to insulin through diet and exercise can be effective. Administration of insulin in patterns that simulate physiologic insulin secretion can also help control weight gain and can be achieved with premixed insulin analogues, e.g., BIAsp 30 and LM. The continuation of metformin after insulin initiation has been associated not only with weight reduction, lower levels of A1C, and lower insulin requirements but also with a higher risk of hypoglycemia [68]. The use of rapid-acting insulin analogues for the mealtime bolus, which when combined with intermediate-acting insulin, can reduce the risk of hypoglycemia and the need for snacks between meals [56]. Nutritional guidance with the purpose of encouraging patients to eat meals low in carbohydrates and to eliminate high-glycemic-index foods altogether from their diet, as well as encouraging patients to maintain a physical activity program, are also helpful in controlling weight gain.

## Conclusions

This nonsystematic review provides a compilation of recent data comparing the efficacy and safety of premixed insulin analogues versus basal insulin analogues used alone or in combination with rapid-acting insulin analogues before meals in patients with T2DM.

Premixed insulin analogues provide both the basal and prandial coverage in a single formulation, which may explain the better glycemic control achieved with BID and TID dosing regimens with premixed analogues compared with a basal only or basal-bolus approach. This regimen also requires fewer BG measurements during the day when compared with basal-bolus therapy, and ensures that the patient receives both basal insulin and bolus insulin at meals with 2 or a maximum of 3 injections per day [13,20].

 Recent data from a meta-analysis [50] and the DURABLE trial [51], suggest that certain patients will benefit more by starting insulin treatment with premixed insulin analogues than others, e.g., patients who have A1C values close to 8.5%, those who have higher elevations in postprandial glucose (difference between preprandial and postprandial >50 mg/dL), those who are less capable of performing several measurements of self-monitored BG during the day for whom a basal-bolus treatment would be more complex to follow, and those who are able to eat regularly. In addition, recent evidence suggests that LM25 provides a similar clinical effect on glycemic control compared with insulin lispro plus glargine in insulin-naïve patients with uncontrolled diabetes on AHAs [55]. In patients who fail to achieve postprandial BG control with a basal insulin, premixed analogues can be an option in order to achieve [13,52] and maintain [51] overall glycemic control. Glycemic control with insulin analogues may be improved using a titration approach [20,56], and treatment needs should be tailored to the individual [9,56].

Individualized glycemic goal setting and establishing when and how to start insulin depend on 2 very important patient-specific aspects: clinical conditions (age, duration of diabetes, presence of microvascular disease and comorbidities, and risk of severe hypoglycemia), and psychosocial/economic context. Glycemic targets can be either more or less stringent from patient to patient [9]. Thus it is important to balance the potential benefits of intensive control with the risk of such intensive targets.

Weight gain, a main limitation of insulin treatment, can significantly affect patient treatment compliance. It is possible to overcome the barriers and main obstacles for patients and physicians when starting insulin treatment by implementing comprehensive education programs for health care professionals [69], patients, and patients’ support networks that can help empower them and clarify concerns and false perceptions related to T2DM treatment [70].

In summary, premixed insulin analogues may mitigate barriers to initiating insulin therapy by providing options for both basal and prandial coverage. It might be a good alternative for people whose control is inadequate with AHAs and/or basal insulin, particularly those patients with postprandial hyperglycemia or high A1C. Similarly, insulin premixes can be the appropriate choice for patients requiring both components of treatment (basal and bolus) but who have restrictions based on the complexity of the basal-bolus regimen. As with any T2DM therapy, insulinization with premixed analogue therapy should adapt to a patient’s lifestyle, hypoglycemia risk, and other factors, and should be individualized.

## Competing interests

MT, MN, and JG do not have any financial competing interests in relation to the manuscript. ME is a former Lilly employee, and CO is a Lilly employee and as such, own Lilly stock. None of the authors have any nonfinancial competing interests in relation to the manuscript.

## Authors’ contributions

The authors (MT, MN, JG, ME, and CO) have made substantive intellectual contributions to a published study and participated sufficiently in the work to take public responsibility for appropriate portions of the content. MT, MN, JG, ME, and CO have made substantial contributions to the acquisition and interpretation of the data in this review; 2) have been involved in drafting the manuscript or revising it critically for important intellectual content; and 3) have given final approval of the version to be published. Each author participated sufficiently in the work to take public responsibility for appropriate portions of the content. All authors read and approved the final manuscript.

## Authors’ information

MT: Head of the discipline of endocrinology, School of Medical Sciences, Universidade Estadual de Campinas.

MN: Endocrinologist, MD, Supervisor of The Medical Service of Endocrinology and Metabolism and Head of the Diabetes Unit of the School of Medicine Universidade de São Paulo.

JG: Professor of Internal Medicine at Universidade Federal do Rio Grande do Sul; Endocrine Division of Hospital de Clínicas de Porto Alegre, Universidade Federal do Rio Grande do Sul.

ME: Endocrinologist, MD. During the preparation of this manuscript, ME was a Diabetes Regional Medical Advisor for Emerging Markets at Eli Lilly.

CO: Endocrinologist, MD, Master of Sciences by discipline Endocrinology FMUSP (School of Medicine of Universidade de Sao Paulo), Diabetes Medical Advisor at Eli Lilly Brazil.
